# Case report: Visual snow as the presenting symptom in multiple evanescent white dot syndrome. Two case reports and literature review

**DOI:** 10.3389/fneur.2022.972943

**Published:** 2022-10-06

**Authors:** Chenyue Hang, Yan Yan

**Affiliations:** ^1^Ottawa-Shanghai Joint School of Medicine, Shanghai Jiao Tong University School of Medicine, Shanghai, China; ^2^Department of Ophthalmology, Renji Hospital, School of Medicine, Shanghai Jiao Tong University, Shanghai, China

**Keywords:** multiple evanescent white dot syndrome (MEWDS), visual snow, chromatopsia, yellow-tinged vision, Chorioretinitis

## Abstract

**Purpose:**

Multiple evanescent white dot syndrome (MEWDS) usually manifests as photopsia, enlarged blind spots, scotomas, and blurred vision, which can be classified into positive and negative visual phenomena. Visual snow and chromatopsia were rarely reported in these patients. Herein, we described two Chinese female patients with MEWDS who initially presented with visual snow, and one of them also had yellow-tinged vision.

**Methods:**

First, we performed the chart review of two patients. Second, we reviewed the English literature for all cases of MEWDS through PubMed until December 2021, using the terms “MEWDS” or “multiple evanescent white dot syndrome.” We concluded on all the reported patients' demographic features and symptoms. The visual acuity of patients with/without positive or negative visual phenomena was compared through one-way ANOVA.

**Results:**

Patient 1: A 27-year-old Chinese woman experienced continuous visual snow and yellow-tinged vision in the right eye for a week. She noticed tiny white and black dots involving the entire visual field and shimmering light inferiorly. Patient 2: A 22-year-old Chinese woman complained of a gray area with continuous visual snow in the temporal visual field of the left eye for 5 days. The ocular examinations, including fundus autofluorescence (FAF), optical coherence tomography (OCT), and indocyanine green angiography (ICGA), confirmed the diagnosis of MEWDS. Their symptoms resolved spontaneously without treatment. We found 60 MEWDS case reports (147 cases) in PubMed. The mean age was 31.2 years old. The mean LogMAR best-corrected visual acuity was 0.35 ± 0.39 at the first visit and 0.01 ± 0.16 at the last visit. The most common symptoms included blurred vision (72.8%), enlarged blind spot (42.2%), photopsia (37.4%), and scotoma (33.3%). We found the patients with only positive visual phenomena had significantly worse visual acuity at the first and last visit than patients with only negative visual phenomena (*p* = 0.008) or the patients with both positive and negative visual phenomena (*p* = 0.026). Four cases similar to visual snow were discovered. Compared to the MEWDS patients without visual snow, the patients with visual snow tend to have a larger proportion of females (*p* = 0.005) and a better visual acuity at the first visit (*p* = 0.007).

**Conclusion:**

Herein, we expand upon the clinical manifestations of MEWDS with visual snow, and the symptoms attributable to visual snow could precede the onset of MEWDS. Neurologists and ophthalmologists should carefully rule out occult chorioretinopathy before diagnosing visual snow syndrome.

## Introduction

Multiple evanescent white dot syndrome (MEWDS) is a self-limited inflammatory chorioretinopathy first described by Jampol et al. (1) and Takeda et al. (2). It is a part of white dot syndrome, which consists of a series of diseases in the presence of multiple discrete white lesions in the deep retina and choroid and shares some overlapping pathology with other white dot syndromes, such as acute posterior multifocal placoid pigment epitheliopathy, multifocal choroiditis and panuveitis, punctate inner choroidopathy, acute zonal occult outer retinopathy, birdshot chorioretinopathy (BCR), and serpiginous choroiditis (3). MEWDS typically manifests as idiopathic, acute, unilateral vision disturbance, such as photopsia, floaters, scotomas, decreased night vision, blurred vision, and visual field loss (3). Under funduscopy, multiple tiny gray-white lesions can be found in the outer retina and retinal pigment epithelium (RPE), extending from perimacular lesions to the mid-peripheral retina. In the acute stage of MEWDS, optical coherence tomography (OCT) may identify the diffuse disruption of the ellipsoid zone (EZ) (4). The exact mechanism of MEWDS remains unknown. It is probably related to immune-related processes like viral infection, especially herpesviridae family and vaccination (5–8) and choriocapillary-based pathologies like hypoperfusion (9), and inflammation of peripapillary circulation (1). Since the disruption of photoreceptors is the primary cause of MEWDS (10, 11), while RPE, choroid, and vitreous are only secondarily involved, MEWDS is named the “common cold of the retina” by Tavallali et al. (12).

MEWDS possesses various visual dysfunctions, which can usually be classified into positive phenomena and negative phenomena (13). Negative phenomena, which indicate the blocking of some part of the real visual images, represent complete visual loss, visual field defects, altered perception of brightness or contrast, altered photosensitivity, nyctalopia, blurring and decreased visual acuity, dyschromatopsia, and abnormal motion perception. Positive visual phenomena are prone to be caused by endogenous neural activity rather than based on external sensory input. They consist of hallucinations (floaters and photopsia), illusions (macropsia, micropsia, metamorphopsia, and palinopsia), and other symptoms like dazzle, glare, and tinting. The mechanism of negative phenomena is mostly the lesions in the visual pathway, while the pathogenesis for positive phenomena remains a mystery. Possible mechanisms contain irritation of the visual pathway, release from visual input, and dysfunction of processing of the visual input (14). In the cases of MEWDS, the patients could have both visual phenomena, suggesting the complex influences of the disease on the visual pathway.

Visual snow is a rare positive visual disturbance describing a phenomenon of numerous flickering dots throughout the visual fields (15). It is different from photopsia, which describes a sudden and brief flash-like visual impairment (16). Visual snow can be idiopathic or secondary to ocular or neurological diseases or hallucinogenic drug usage (17). It usually appears with other positive visual phenomena like palinopsia and floaters, known as visual snow syndrome (VSS). Different from visual snow, VSS refers to a syndrome with more specific diagnostic criteria and other associated features (18). Klein et al. illustrate that VSS is a network disorder characterized by excessive activation of the visual cortices with irrelevant internal and external stimulus, which suggests the dysfunction in “filtering” and prioritizing stimuli (19). Visual snow may be an atypical presentation in patients with uveitis and retinal diseases. In the previously reported cases, visual snow could present in BCR, another member of white dot syndromes (20). Nevertheless, there were no reports of visual snow in other white dot syndromes. MEWDS could represent neglectable dots under fundoscopy with an insidious onset, recover spontaneously in a short time, and thus be misdiagnosed as VSS with inadequate tests. Herein, we reported two patients presenting with visual snow, who was finally diagnosed with MEWDS. In addition, yellow-tinged vision, a kind of chromatopsia, was also noted in one patient.

## Case descriptions

### Case 1

**Figure 1:**
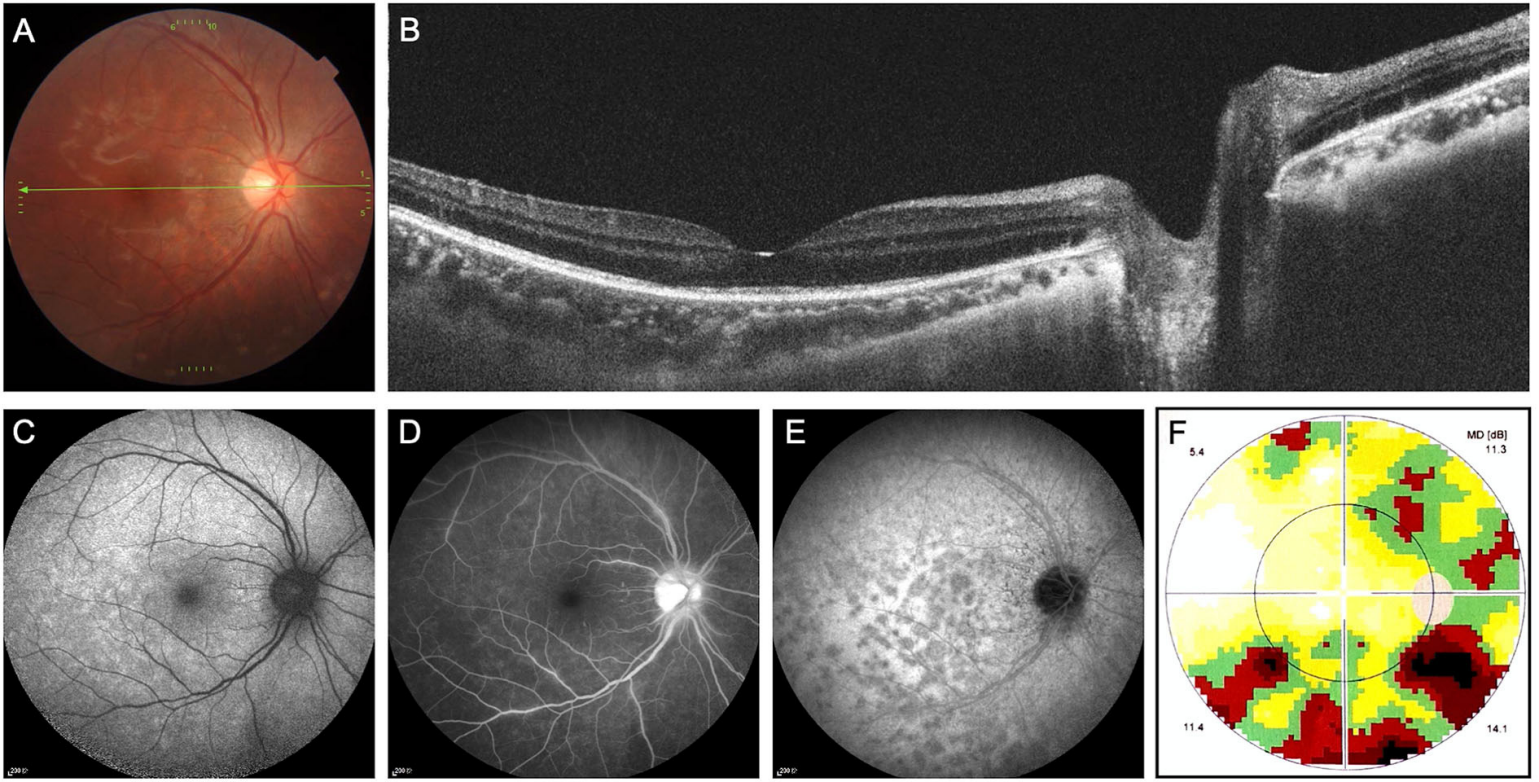
Multimodal imaging of multiple evanescent white dot syndrome (patient 1). **(A)** Color fundus imaging of the right eye showed subtle multifocal white dotted lesions in the posterior pole. **(B)** Optical coherence tomography showed discontinuities and attenuation of external limiting membrane and ellipsoid zone. **(C)** Fundus autofluorescence revealed scattered hyper-autofluorescence. **(D)** Fundus angiography showed punctate hyperfluorescence and staining of the venous wall. **(E)** Indocyanine green angiography (ICGA) demonstrated numerous hypocyanescent dots and spots, which outnumbered the lesions. **(F)** The static visual field test revealed blind spot enlargement and inferior defects in the right eye.

A 27-year-old Chinese woman experienced continuous visual snow and yellow-tinged vision in the right eye for a week. She complained of tiny white and black dots involving the entire visual field of the right eye and shimmering light in the inferior visual field, especially in the dim light. No flashes of light across the visual field were witnessed by her. Her past medical history was unremarkable except for myopia of −3 diopters in both eyes, and she did not take any medication or illicit drugs. On initial examination, her best-corrected visual acuity (BCVA) and color vision were normal. The slit-lamp examination was normal in both eyes. A mild right relative afferent pupillary defect (RAPD) was noted. Color fundus imaging of the right eye showed subtle multifocal white dotted lesions in the posterior pole (Figure 1A). OCT showed discontinuities and attenuation of external limiting membrane and ellipsoid zone (Figure 1B). Fundus autofluorescence (FAF) revealed scattered hyper-autofluorescence (Figure 1C). Fluorescein angiography (FA) showed punctate hyperfluorescence and staining of the venous wall (Figure 1D). Indocyanine green angiography (ICGA) demonstrated numerous hypocyanescent dots and spots, which outnumbered the lesions (Figure 1E). The visual field test revealed blind spot enlargement and inferior defects in the right eye (Figure 1F). The clinical presentations and multimodal imaging were consistent with MEWDS in the right eye. After a three-month follow-up without treatment, the lesions disappeared, and her symptoms gradually resolved.

### Case 2

**Figure 2:**
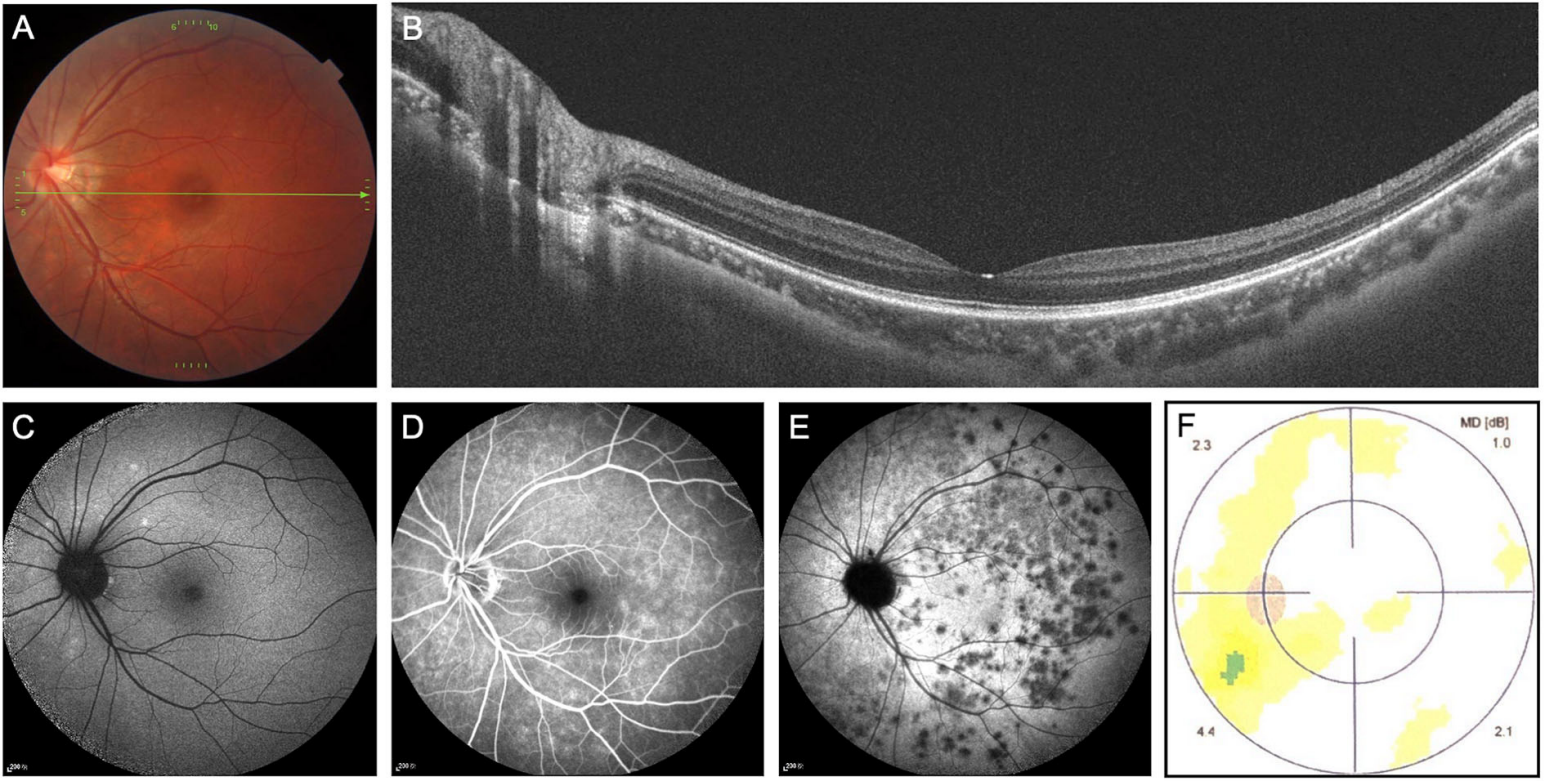
Multimodal imaging of multiple evanescent white dot syndrome (patient 2). **(A)** Color fundus imaging of the left eye showed normal fundus. **(B)** Optical coherence tomography showed a diminished ellipsoid zone in the peripapillary region. **(C)** Fundus autofluorescence (FAF) revealed several spotty hyper-autofluorescence. **(D)** Fundus angiography showed wreath-like hyperfluorescence in the late phase. **(E)** Indocyanine green angiography demonstrated numerous hypocyanescent dots and spots, which outnumbered the lesions on FAF. **(F)** The static visual field test revealed enlargement of the blind spot in the left eye.

A 22-year-old Chinese woman complained of a gray area with continuous visual snow in the temporal visual field of the left eye for 5 days. She also had blurry vision in that part of the visual field. She did not notice any photopsia. Her past medical history included mild myopia in both eyes, and she did not take any medication or illicit drugs. On initial examination, her BCVA was 20/20, and color vision was normal. There were no signs of anterior or posterior segment inflammation. Color fundus imaging of the left eye showed normal fundus (Figure 2A). OCT showed a diminished ellipsoid zone in the peripapillary region (Figure 2B). FAF revealed several spotty hyper-autofluorescence (Figure 2C). FA showed wreath-like hyperfluorescence in the late phase (Figure 2D). ICGA demonstrated numerous hypocyanescent dots and spots, which outnumbered the lesions on FAF (Figure 2E). The visual field test demonstrated blind spot enlargement in the left eye (Figure 2F). The patient was diagnosed with visual snow and enlargement of the blind spot in the left eye secondary to MEWDS. Without any treatment, the symptoms resolved on their own. Fundus examinations showed healthy retina on OCT and FAF at a two-month follow-up.

## Literature review

### Materials and methods

We searched all the reported MEWDS cases on PubMed until December 2021. The search strings used were “MEWDS” or “multiple evanescent white dot syndrome”. We recorded the basic information and clinical manifestations of all the cases. Since the manifestations are in our review, we divided the symptoms of MEWDS into three main groups: (1) Positive visual phenomena: photopsia/flashes of light/shimmer, floaters, and metamorphopsia; (2) Negative symptoms: blurred vision, enlarged blind spot, visual field loss, scotoma/black spots, gauze/gray haze or area, purple haze, color vision defect/dyschromatopsia, sensitivity loss, and night blindness; (3) Other symptoms like periorbital pain, photophobia, headache, and RAPD. Accordingly, we classified patients into three groups: (1) patients with positive symptoms (with or without other symptoms); (2) patients with negative symptoms (with or without other symptoms); (3) patients with both positive and negative symptoms (with or without other symptoms).

## Results

The initial search returned 88 correlative articles. Nine manuscripts were reviewed. Nineteen of them had a blurry description of clinical symptoms and basic information. Finally, 60 related case reports were found, and the clinical presentations of 147 cases (No.1–No.147) were clearly depicted. We use the data of these 147 cases for analysis. According to the classification method, we concluded the sex ratio, mean age, mean LogMAR visual acuity of the affected eye at the first and last visit, and symptoms of each group in Table 1. The patients had a mean age of 31.2 years with a standard deviation of 11.3 years, showing a female-to-male ratio of 4.88. In the affected eyes, the mean LogMAR BCVA was 0.35 ± 0.39 at the first visit and 0.01 ± 0.16 at the last visit. The most common symptoms included blurred vision (72.8%), photopsia (37.4%), enlarged blind spot (42.2%), and scotoma (33.3%). Through one-way ANOVA, we found that the patients in group 1 had significantly worse visual acuity at the first and last visit than group 2 (*p* = 0.008) and group 3 (*p* = 0.026), while no difference could be drawn between group 2 and group 3. No other significant discrepancy could be drawn from Table 1.

**Table 1:** Characteristics of three groups of patients with multiple evanescent white dot syndrome in the literature.

	**Group 1 (*n =* 10) patients with positive symptoms (±other symptoms)**	**Group 2 (*n =* 80) patients with negative symptoms (±other symptoms)**	**Group 3 (*n =* 57) patients with both positive and negative symptoms (±other symptoms)**	**Total (*n =* 147)**
Sex, female/male	10 F	63 F / 17 M	49 F / 8 M	122 F / 25 M
Age at onset, mean (SD), years	28.7 (9.87)	30.3 (10.8)	33.1(12.2)	31.2 (11.3)
BCVA in the affected eye at the first visit, logMAR(SD) [Snellen]	0.16 (0.37)	0.38 (0.38)	0.34 (0.39)	0.35 (0.39)
BCVA in the affected eye at the last visit, logMAR(SD) [Snellen]	−0.07 (0.05)	0.02 (0.14)	0.03 (0.20)	0.01 (0.16)
Symptoms, %	Photopsia/Flashes of light/Shimmer 40.0%; Metamorphopsia 60.0%	Blurred vision 88.8%; Enlarged blind spot 45.0%; Visual field loss 13.8%; Scotoma/Black spots 27.5%; Purple haze 1.3%; Night blindness 1.3%; Periorbital pain 1.3%; Photophobia 1.3%	Photopsia/Flashes of light/Shimmer 89.5%; Metamorphopsia 7.0%; Floaters 10.5%; Blurred vision 63.2%; Enlarged blind spot 45.6%; Visual field loss 8.8%; Scotoma/Black spots 47.4%; Gauze/Gray haze/Gray area 7.0%; Defect color vision/ Dyschromatopsia 5.3%; Sensitivity loss 5.3%; Periorbital pain 3.5%; Headache 8.8%; RAPD 5.3%	Photopsia/Flashes of light/Shimmer 37.4%; Metamorphopsia 6.8%; Floaters 4.1%; Blurred vision 72.8%; Enlarged blind spot 42.2%; Visual field loss 10.9%; Scotoma/Black spots 33.3%; Gauze/Gray haze/Gray area 2.7%; Purple haze 0.7%; Defect color vision/ Dyschromatopsia 2.0%; Sensitivity loss 2.0%; Night blindness 0.7%; Periorbital pain 2.0%; Headache 3.4%; RAPD 2.0%; Photophobia 0.7%

BCVA, best-corrected visual acuity; SD, standard deviation; logMAR, logarithmic minimum angle of resolution; RAPD, relative afferent pupillary defect.

The symptoms of most cases would disappear spontaneously, and most of those symptomatic cases would resolve after using corticosteroids. However, 28 (19%) cases had long-lasting visual disturbances and complications or relapses (Supplementary Table). Eight cases had MEWDS relapses, and 12 still had remaining or worsening symptoms like enlarged blind spot and visual field defect even after treatments. In comparison, eight cases experienced severe complications later, like optic neuritis (ON), type 2 choroidal neovascularization (CNV), acute zonal occult outer retinopathy (AZOOR), vitreous detachment, and idiopathic posterior uveitis.

We also find a few detailed and notable descriptions of MEWDS that could not simply be explained by the typical symptoms above (Table 2). In case No. 93 (10), a 39-year-old woman described a polka-dot visual field defect, floaters, and blurred vision. The disrupted ellipsoid zone recovered after 16 weeks. However, multifocal electroretinography uncovered a reduced temporal response, corresponding to a persistent enlarged blind spot. In case No. 117 (21), a 22-year-old woman noted multiple foci of photopsia and scotomas in the central visual field. An acute macular neuroretinopathy was diagnosed based on multiple round red-orange lesions at the level of the outer retina in the central macular area. She was given prednisone, and all symptoms improved except paracentral scotomas. Five years later, she was returned with even more severe and similar symptoms as before after an upper respiratory tract infection, with all the examinations suggesting MEWDS. In case No. 133 (22), a 30-year-old woman encountered a dazzling sensation of light accompanied by numerous dark spots after falling to the ground. FAF, OCT, and ICGA results all supported the diagnosis of MEWDS. After 2 months, the patient had intermittent photopsia with improving visual blur. In case No. 136 (23), a 34-year-old Hispanic woman with myopia was found to have peripheral flickering lights and central dots. Her symptoms spontaneously resolved after 2 weeks, leaving an orange foveal granularity with patches of brown granular changes temporal to the fovea. All four patients had similar presentations and examination results to other MEWDS. Three patients had probable visual snow in partial visual field similar to our patients. One patient was not mentioned to have a pan-filed or partial-field visual disturbance. Compared to the other 143 collected cases without visual snow, the cases with visual snow (four cases in previous reports and our two cases) had a higher proportion of females (*p* = 0.005) and a better visual acuity at the first visit (*p* = 0.007). No differences were found between the four previous cases and our cases, possibly due to the limited data size. Based on these cases and our patients, we now raise the idea that visual snow could be an atypical presentation of MEWDS and the mechanism below is worth exploring.

**Table 2:** The basic information of multiple evanescent white dot syndrome patients with suspected secondary visual snow in the literature and in our center.

**Case No**.	**Author, year**	**Age, sex**	**Ethnic group**	**BCVA in the affected eye (at first vist, at last visit)**	**Symptoms**	**Treatment, outcome**
93	Li et al. (10)	39, Female	NA	20/30, 20/30, OD	Blurred vision, a polka-dot visual field defect, and floaters	NA, symptoms disappear except enlargement of blind spot
117	Gass et al. (21)	22, Female	NA	20/80, normal, OS	Multiple foci of photopsia (central visual field), central scotomas, multiple negative scotomas, loss of vision	prednisone, persistent temporal scotoma for several years, while had further visual disturbance after 5 years.
133	Vasseur et al. (22)	30, Female	NA	20/20, NA, OD	A dazzling sensation of light with numerous dark spots (temperal visual field)	NA, visual blur improved while intermittent photopsia remained after 2 months.
136	Bhakhri et al. (23)	34, Female	Hispanic	20/20, 20/20, OD	Flickering light (peripheral visual field) and dots (central visual field)	None, spontaneous and complete remission of presentation except an orange foveal granularity
1^*^	Case 1	27, Female	Chinese	20/20, 20/20, OD	Visual snow thoughout the visual field and inferior shimmering lights and yellow-tinged vision	None, symptoms gradually resolved.
2^*^	Case 2	22, Female	Chinese	20/20, 20/20, OD	a gray area with continuous visual snow temporally, and blurry vision	None, symptoms gradually resolved.

* The cases reported in this article.

NA, not available.

## Discussion

MEWDS is an occult inflammatory chorioretinopathy. Modern ophthalmic modalities, such as OCT, ICGA, and FAF, are often more helpful in detecting lesions than fundus ophthalmoscopy or fundus photograph. It may present positive and/or negative visual phenomena. As far as we know, visual snow has not been specifically described in patients with MEWDS. We described two Chinese patients with visual snow as the presenting symptoms of MEWDS. In the literature search, we also found some noteworthy manifestations like “multiple foci of photopsia and scotomas” (21), “flickering lights and spots” (23), “a polka-dot visual field defect” (10), and “a dazzling sensation of light with numerous dark spots” (22), which may suggest the presence of visual snow in these MEWDS patients. Owing to the atypical presentation and the lack of the other ocular symptoms of visual snow syndrome, we consider these visual snow symptoms as part of MEWDS manifestations instead of the presentation of VSS. No significant difference was found between the four previous cases and our patients. However, when comparing all six cases with visual snow to the other MEWDS cases without visual snow, we found that a higher proportion of females and better initial VA was related to the presence of visual snow.

Visual snow is either a positive visual disturbance based on a retinal pathology or a cortical phenomenon (24). In MEWDS cases, the pathogenesis of visual snow is prone to be retinal origin and is similar to that in BCR, which is also classified as white dot syndrome (20, 25). Different from MEWDS, BCR will progress and lead to irreversible visual loss and severe tissue damage (26). Kuiper et al. speculated that the anti-viral immune response would probably attack eye tissues in these patients (27). Since MEWDS is also prone to occur following viral infection and vaccination (28), a credible suppose could be raised that MEWDS is also caused by an autoimmune cross-reaction between viral antigens and ocular antigens. Furthermore, the direct and indirect immune attack of the retinal tissues in MEWDS and BCR leads to visual snow in both groups of patients.

Central serous retinopathy and retinitis pigmentosa had been reported as the causes of visual snow. The ocular fundus examination and ancillary investigations will help make differential diagnoses (17, 29). There is no association reported in autoimmune or paraneoplastic retinopathy. However, these conditions mimic white dot syndromes and may present with positive visual phenomenon such as photopsia. The diagnosis is often challenging and is largely based on the existence of antiretinal antibodies in the serum along with the examinations (30).

Importantly, patients sometimes mischaracterize the symptoms, so those symptoms that mimic vs. in the reported cases and our cases could be attributable to other ocular symptoms. As a result, it is essential for clinicians to differentiate visual snow symptoms (continuous flickering tiny dots over the entire visual field) from other symptoms like photopsia and provide guidance for patients to help them give more accurate descriptions. Clinicians also should perform an accurate examination and follow-up in patients with suspected VS. There are several red flags in patients with visual snow: new-onset rather than preexisting visual snow, visual snow in partial rather than the whole visual field, unilateral rather than bilateral visual snow, any neurological deficit, and any vision change (including visual or visual field loss). Those red flags alert the clinicians to perform more extensive examinations to rule out ophthalmic or neurological diseases. After asking for a thorough medical history and performing neurological examination and ocular examination, clinicians also should perform brain MRI with DWI or EEG to exclude neurological diseases and retinal imaging/ OCT /electrophysiological testing to exclude ocular diseases (17). Table 3 lists the recommendations of ophthalmology review and investigations as part of the workup in patients with visual snow. We did not perform electroretinography (ERG) or multifocal ERG in these two patients. Further studies should look for whether the areas of local abnormalities of mfERG correspond to the areas of visual snow presented within.

**Table 3:** Recommendations of ophthalmology review and investigations as part of the workup in patients with visual snow.

Presentations	If the patients have blurred vision, vision loss, visual field defect, acquired color vision defect, metamorphopsia, floaters, or photopsia
Ocular examinations	Any of the following signs are detected: Cells in aqueous humor and vitreous Optic disc swelling or atrophy Retinal or choroidal lesions
Ancillary investigations	Automated perimetry, OCT, and FAF (FFA, ERG, mfERG, and VEP if needed)

ERG, electroretinography; FAF, fundus autofluorescence; FFA, fundus fluorescein angiography; ICGA, indocyanine green angiography; mfERG, multifocal electroretinography; VEP, visual evoked potential.

## Conclusion

In conclusion, our case reports demonstrate that patients with MEWDS can initially present visual snow without noticeable vision loss, that is, the symptoms attributable to visual snow could precede the onset of a MEWDS. Therefore, neurologists and ophthalmologists should be aware of white dot syndromes in these patients with visual snow and perform an accurate examination and follow-up to ensure there is no occult chorioretinopathy.

## Data availability statement

The raw data supporting the conclusions of this article will be made available by the authors, without undue reservation.

## Ethics statement

The studies involving human participants were reviewed and approved by Renji Hospital, Shanghai Jiao Tong University, School of Medicine. The patients/participants provided their written informed consent to participate in this study. Written informed consent was obtained from the individual(s) for the publication of any potentially identifiable images or data included in this article.

## Author contributions

YY examined the patients, established the diagnosis, composed the figures, and revised the manuscript. CH wrote the whole manuscript and searched the literature. Both authors contributed to the article and approved the submitted manuscript.

## Conflict of interest

The authors declare that the research was conducted in the absence of any commercial or financial relationships that could be construed as a potential conflict of interest.

## Publisher's note

All claims expressed in this article are solely those of the authors and do not necessarily represent those of their affiliated organizations, or those of the publisher, the editors and the reviewers. Any product that may be evaluated in this article, or claim that may be made by its manufacturer, is not guaranteed or endorsed by the publisher.
