# Supplementary material for: Case report: Visual snow as the presenting symptom in multiple evanescent white dot syndrome. Two case reports and literature review
Source: Front Neurol. 2022 Oct 6;13:972943. doi: 10.3389/fneur.2022.972943 (PMC9582439; doi:10.3389/fneur.2022.972943)
Supplement: Supplementary file 1 [file Table_1.docx]

Supplementary Material

# Supplementary Table

**Table** The information of MEWDS patients with remained symptoms, relapses, or complications.

| Case No. | Author, year | Age, Sex | Ethnic group | BCVA in the  affected eye at the first visit | Symptoms (Group) | Type | Treatment, Outcome |
| --- | --- | --- | --- | --- | --- | --- | --- |
| 2 | Conrady^(1)^, 2021 | 41, Female | NA | 0.8, OS | Shimmering, photopsias (Group 1) | Complication | Prednisone, symptoms resolved but ﻿after 4 years the patient reported pain with eye movements, vision loss, worsening color vision, and diagnosed as ON. |
| 13 | ﻿Tsai^(2)^, 1994 | 24, Female | NA | 0.4, OD | Crescentic paracentral scotoma, multiple round spots, numerous small paracentral scotomas, decreased central vision, enlarged blind spots (Group 2) | Relapses | None, 7 episodes of relapse MEWDS characterized by visual blurring and enlarged blind spot. |
| 44 | Li^(3)^, 2009 | 33, Male | NA | 1.0, OD | Blurred vision, enlarged blind spot (Group 2) | Remained symptoms | None, slight enlarged blind spot. |
| 51 | ﻿Kuznetcova^(4)^, 2012 | 40, Female | NA | 0.8, OS | Paracentral scotoma (Group 2) | Relapses | None, after 1 year ﻿the patient was seen again for the same subjective scotoma in her left eye accompanied by photopsias. BCVA was 1.0 OD and 0.9 OS. |
| 52 | ﻿Kuniyoshi^(5)^, 2016 | 25, Female | Japanese | 1.5, OS | Visual field abnormality, enlarged blind spot (Group 2) | Remained symptoms | None, visual field defect is still present in bright areas after 4 years. |
| 58 | Tsai^(2)^, 1994 | 24, Female | NA | 0.07, OS; 0.4, OD | Vision loss in both eyes (Group 2) | Relapses | None, three recurrence episodes in OD and one in OS. |
| 68 | Chen^(6)^, 2017 | 29. Female | NA | 0.13, OS | Decrased vision acuity (Group 2) | Complication | Anti-VEGF injections, ﻿a subretinal hemorrhage near the foveal exudate showed well-defined hyperfluorescent leakage on FA consistent with Type 2 neovascularization. |
| 69 | Chen^(6)^, 2017 | 29, Male | NA | 0.05, OD | Decrased vision acuity (Group 2) | Complication | None, ﻿a small area of Type 2 neovascularization. |
| 82 | Feigl^(7)^, 2002 | 23, Female | NA | 0.8, OS | Blurred vision (Group 2) | Remained symptoms | ﻿None, still suffered from visual impairment but showed improved angiographic findings and stable, slightly decreased and subnormal electroretinographic changes after 6 weeks’ follow up. |
| 89 | Fine^(8)^, 2009 | 29, Female | Hispanic | 0.2, OD | Blurry rision, periorbital pain, enlarged blind spot, marked right temperal visual field loss (Group 2) | Complication | None, developed progressive visual field loss OD after 14 months and diagnosed as AZOOR. |
| 90 | Feigl^(7)^, 2002 | 25, Female | NA | 0.5, OS | Visual disturbances, photophobia, blurred vision, multiple scotomas (Group 2) | Remained symptoms | ﻿None, still suffered from visual impairment but showed improved angiographic findings and stable, slightly decreased and subnormal electroretinographic changes after 6 weeks’ follow up |
| 93 | Li^(3)^, 2009 | 39, Female | NA | 1.2, OD | Blurred vision, enlarged blind spot, a polka-dot visual field defect, floaters(Group 2) | Remained symptoms | None, slight enlarged blind spot |
| 103 | Penha^(9)^, 2011 | 32, Female | NA | 0.625, OS | Photopsia, vision loss (Group 3) | Relapses | ﻿None, thirty days later, the patient recalled sporadic episodes of photopsia |
| 108 | Haw^(10)^, 2020 | 24, Female | Chinese | 1.2, OS | Flashes of light, blurred vision, a superior and temporal paracental scotoma, enlarged blind spot (Group 3) | Relapses | None, two years later the patient ﻿experienced persistent flashes of light with acute onset of blurry vision in her right eye following an upper respiratory tract infection. |
| 110 | Oh^(11)^, 2001 | 27, Female | NA | 0.67, OD | Photopsia and blurred vision, enlarged blind spot (Group 3) | Remained symptoms | ﻿None, a minimal residual enlargement of the blind spot |
| 117 | Gass^(12)^, 1989 | 22, Female | NA | 0.67, OS | Multiple foci of photopsia, central scotomas, multiple negative scotomas, loss of vision (Group 3) | Remained symptoms | Prednisone, persistent temporal scotoma for several years, while had further visual disturbance after 5 years. |
| 118 | Gass^(12)^, 1989 | 30, Female | NA | 0.25, OS | Blurred vision, large temperal scotoma, shimmering lights emanating from the scotoma in the left eye, enlarged blind spot, multiple paracentral scotomas | Remained symptoms | Prednisone, his symptoms were unchanged except partical resolution of the blind spot enlargement |
| 119 | Labriola^(13)^, 2016 | 24, Female | NA | 0.67, OD | Decreased vision centrally, enlarged blind spot, photopsias | Relapses | None, 3 relapse and each flare presented with decreased vision centrally and enlarged blind spot and photopsias, in between flares vision recovered first, then the blind spot slowly improve, photopsias persisted for months prior to resolution |
| 120 | Conrady^(1)^, 2021 | 22, Female | NA | 0.07, OD | Dark spot in the peripheral visual field, persistent flickering photopsias (Group 3) | Relapses | None, the patient developed similar episode OS. |
| 130 | Yang^(14)^, 2018 | 33, Female | Chinese | 1.0, OS | Large paracentral scotoma, photopsias (Group 3) | Remained symptoms | Retrobulbar triamcinolone acetonide, partially resolved at 8 weeks |
| 133 | Vasseur^(15)^, 2020 | 30, Female | NA | 1.0, OD | Intermittent photopsia described as a dazzling sensation of light with numerous dark spots that were predominant in the temporal visual field of the affected eye，enlarged blind spot (Group 3) | Remained symptoms | None, two months later, the patient noted that her visual blur improved, but still had intermittent photopsia |
| 140 | ﻿Barile^(16)^, 2017 | 64, Male | NA | 0.05, OD | ﻿Central visual loss with intermittent photopsia, absolute scotoma with adjacent relative scotomas, decreased vision, a central scotoma with shimmering, gauze effect of the vision, a trace afferent pupillary defect (Group 3) | Complication | Prednisone and ﻿aflibercept, ﻿the retina has remained unchanged during further follow-up, specifically without evolution to further retinal lesions over 2 years. The right eye subsequently experienced a vitreous separation without sequelae. The acuity has fluctuated between 20/250 and 20/400 during this time. |
| 141 | Fine^(8)^, 2009 | 35, Female | Hispanic | 1.0, OD | Photopsias, a temporal blind spot, headache, temporal field loss (Group 3) | Complication | None, further temporal field loss and diagnosed as AZOOR. |
| 142 | Ogino^(17),^ 2014 | 16, Female | NA | 1.2, OS | Photopsia, sensitivity loss in the temporal inferior visual field, atypical scotoma, headache (Group 3) | Remained symptoms | ﻿Prednisolone and ﻿betamethasone, scotomata enlargement |
| 143 | Conrady^(1)^, 2021 | 36, Female | NA | 0.5, OS | ﻿Acute vision loss and pain with eye movements, ﻿subjective color desaturation, vision loss, "ripples" in the vision (Group 3) | Complication | None, twelve months later she was diagnosed with idiopathic posterior uveitis. |
| 144 | ﻿Tsai^(2)^, 1994 | 21, Female | NA | NA | Photopsia, scotoma, pain on eye movement, enlarged blind spot, retrobulbar headache, a trace right afferent pupillary defect (Group 3) | Relapses | Corticosteriods, 7 episodes of MEWDS. |
| 146 | Fine^(8)^, 2009 | 51, Male | NA | Counting fingers, OD | Photopsia, headache, enlarged blind spot, marked temporal visual field loss (Group 3) | Complication | None, eleven months later the patient developed a AZOOR. |
| 147 | Yang^(14)^, 2018 | 16, Female | Japanese | 1.25, OS | Headache, Photopsias, peripheral vision loss (Group 3) | Remained symptoms | Betamethasone and anti-histamine, Retinal lesions resolved at two months; worsening peripheral vision loss for 2 years. |

NA, not available; ON, optic neuritis; VEGF, ﻿vascular endothelial growth factor; FA, ﻿fluorescein angiography; MEWDS, multiple evanescent white dot syndrome; AZOOR, ﻿acute zonal occult outer retinopathy.

**Reference**

1. Conrady CD, Sassalos T, Cornblath WT, Zacks DN, Johnson MW. Temporally Independent Association of Multiple Evanescent White Dot Syndrome and Optic Neuritis. *Graefes Arch Clin Exp Ophthalmol* (2021) 259(9):2807-11. Epub 2021/05/30. doi: 10.1007/s00417-021-05249-2.

2. Tsai L, Jampol LM, Pollock SC, Olk J. Chronic Recurrent Multiple Evanescent White Dot Syndrome. *Retina* (1994) 14(2):160-3. Epub 1994/01/01. doi: 10.1097/00006982-199414020-00009.

3. Li D, Kishi S. Restored Photoreceptor Outer Segment Damage in Multiple Evanescent White Dot Syndrome. *Ophthalmology* (2009) 116(4):762-70. Epub 2009/04/07. doi: 10.1016/j.ophtha.2008.12.060.

4. Kuznetcova T, Jeannin B, Herbort CP. A Case of Overlapping Choriocapillaritis Syndromes: Multimodal Imaging Appraisal. *J Ophthalmic Vis Res* (2012) 7(1):67-75. Epub 2012/06/28.

5. Kuniyoshi K, Sakuramoto H, Sugioka K, Matsumoto C, Kusaka S, Shimomura Y. Long-Lasting, Dense Scotoma under Light-Adapted Conditions in Patient with Multiple Evanescent White Dot Syndrome. *Int Ophthalmol* (2016) 36(4):601-5. Epub 2015/12/19. doi: 10.1007/s10792-015-0163-6.

6. Chen KC, Marsiglia M, Dolz-Marco R, Zahid S, Mrejen S, Pulido JS, et al. Foveal Exudate and Choroidal Neovascularization in Atypical Cases of Multiple Evanescent White Dot Syndrome. *Retina* (2017) 37(11):2025-34. Epub 2017/01/19. doi: 10.1097/iae.0000000000001486.

7. Feigl B, Haas A, El-Shabrawi Y. Multifocal Erg in Multiple Evanescent White Dot Syndrome. *Graefes Arch Clin Exp Ophthalmol* (2002) 240(8):615-21. Epub 2002/08/23. doi: 10.1007/s00417-002-0478-7.

8. Fine HF, Spaide RF, Ryan EH, Jr., Matsumoto Y, Yannuzzi LA. Acute Zonal Occult Outer Retinopathy in Patients with Multiple Evanescent White Dot Syndrome. *Arch Ophthalmol* (2009) 127(1):66-70. Epub 2009/01/14. doi: 10.1001/archophthalmol.2008.530.

9. Penha FM, Navajas EV, Bom Aggio F, Rodrigues EB, Farah ME. Fundus Autofluorescence in Multiple Evanescent White Dot Syndrome. *Case Rep Ophthalmol Med* (2011) 2011:807565. Epub 2011/01/01. doi: 10.1155/2011/807565.

10. Haw YL, Yu TC, Yang CS. A Care-Compliant Article: A Case Report of Possible Association between Recurrence of Multiple Evanescent White Dot Syndrome and the Herpesviridae Family. *Medicine (Baltimore)* (2020) 99(15):e19794. Epub 2020/04/14. doi: 10.1097/md.0000000000019794.

11. Oh KT, Folk JC, Maturi RK, Moore P, Kardon RH. Multifocal Electroretinography in Multifocal Choroiditis and the Multiple Evanescent White Dot Syndrome. *Retina* (2001) 21(6):581-9. Epub 2002/01/05. doi: 10.1097/00006982-200112000-00004.

12. Gass JD, Hamed LM. Acute Macular Neuroretinopathy and Multiple Evanescent White Dot Syndrome Occurring in the Same Patients. *Arch Ophthalmol* (1989) 107(2):189-93. Epub 1989/02/01. doi: 10.1001/archopht.1989.01070010195021.

13. Labriola LT, Legarreta AD, Legarreta JE, Nadler Z, Gallagher D, Hammer DX, et al. Imaging with Multimodal Adaptive-Optics Optical Coherence Tomography in Multiple Evanescent White Dot Syndrome: The Structure and Functional Relationship. *Retin Cases Brief Rep* (2016) 10(4):302-9. Epub 2016/01/07. doi: 10.1097/icb.0000000000000271.

14. Yang JS, Chen CL, Hu YZ, Zeng R. Multiple Evanescent White Dot Syndrome Following Rabies Vaccination: A Case Report. *BMC Ophthalmol* (2018) 18(1):312. Epub 2018/12/12. doi: 10.1186/s12886-018-0968-y.

15. Vasseur V, Arej N, Alonso AS, Lafolie J, Philibert M, Vignal-Clermont C, et al. Spectralis High Magnification Module Imaging in a Case of Multiple Evanescent White Dot Syndrome. *Am J Ophthalmol Case Rep* (2020) 19:100727. Epub 2020/05/19. doi: 10.1016/j.ajoc.2020.100727.

16. Barile GR, Harmon SA. Multiple Evanescent White Dot Syndrome with Central Visual Loss. *Retin Cases Brief Rep* (2017) 11 Suppl 1:S219-s25. Epub 2016/11/09. doi: 10.1097/icb.0000000000000467.

17. Ogino K, Kishi S, Yoshimura N. Multiple Evanescent White Dot Syndrome after Human Papillomavirus Vaccination. *Case Rep Ophthalmol* (2014) 5(1):38-43. Epub 2014/04/08. doi: 10.1159/000358870.
